# Multi-site microbiota crosstalk in the postmenopausal: from dysbiosis mechanisms to precision interventions

**DOI:** 10.3389/fmicb.2026.1702700

**Published:** 2026-02-19

**Authors:** Jiaqing Fang, Xiaobo He, Junjun Zhou

**Affiliations:** Department of Obstetrics and Gynaecology, Affiliated Women and Children's Hospital of Ningbo University, Ningbo, China

**Keywords:** metabolic syndrome, microbiome, microbiota modulation, mood disorders, osteoporosis, postmenopause

## Abstract

Persistent estrogen deficiency in postmenopause reshapes the gut–vaginal–urinary ecosystems, contributing to multisystem dysfunction through interconnected dysbiosis. Cross-niche microbial interactions amplify systemic risks: gut-derived toxins are linked to vascular inflammation, vaginal dysbiosis facilitates urinary pathogen colonization, and bile acid disruption impairs bone–immune homeostasis. This review synthesizes current mechanistic evidence illustrating how microbial networks propagate postmenopausal comorbidities. We propose the framework of “Postmenopausal Microbiota Network Medicine” which integrates microbial dynamics with host omics to transition from symptomatic management toward root-cause interception by strengthening microbial network resilience.

## 1 Introduction

Postmenopause constitutes a persistent state of estrogen deficiency following reproductive senescence, which contributes to multisystem dysfunction through progressive microbial dysregulation. This phase markedly elevates the risk of osteoporosis, cardiovascular disease (CVD), metabolic syndrome, and genitourinary disorders, collectively impairing quality of life (Mishell, 1989; Fitzpatrick, 1999; Tiwari et al., 2025; Hirahatake et al., 2019).

Evidence indicates that sustained estrogen loss remodels the gut–vaginal–urinary ecosystems, triggering pathogenic cross-communication: gut dysbiosis amplifies endotoxin leakage and atherogenic metabolites, vaginal *Lactobacillus* depletion compromises urethral barrier integrity and facilitates urinary pathogen colonization—and bile acid disruption impairs osteoimmune homeostasis (Sapra et al., 2025; Parsons and Schmidt, 1982; Valiquette, 2001). Crucially, the gut microbiota—as a systemic orchestrator—propagates dysbiotic networks implicated in chronic inflammation and may contribute to tissue damage (Adak and Khan, 2019; Järbrink-Sehgal and Andreasson, 2020; Fan and Pedersen, 2021).

The mechanisms underlying this multi-niche dysbiosis are multifactorial. Estrogen deficiency directly impairs glycogen deposition in the vaginal epithelium, reducing the primary metabolic substrate for glycogen-metabolizing *Lactobacillus* species. Concurrently, systemic estrogen loss alters gut barrier function and immune homeostasis, facilitating translocation of pro-inflammatory microbial products. These changes collectively create environments conducive to the expansion of pathobionts while diminishing protective microbiota across ecological niches.

The dynamic changes across ecosystems—specifically, gut metabolites exacerbating vascular endothelial cell pyroptosis and urinary tract pathogen invasion of atrophied epithelium—may represent one of the pathogenic mechanisms underlying postmenopausal diseases (Brunt et al., 2019; Muttiah and Hanafiah, 2025; Qiao et al., 2022). Precision strategies target microbial network resilience through synchronized interventions: multi-niche probiotic consortia, Fecal Microbiota Transplantation (FMT) tailored to estrogen-deficient phenotypes (Cross et al., 2024), and metabolite supplementation.

Elucidating microbiota-chronic disease axes requires multilevel integration. Future research should prioritize combinatorial therapeutics and recalibrate to optimize healthspan.

## 2 Literature search and scope

This narrative review was conducted to synthesize current evidence on the role of gut, vaginal, and urinary microbiota in postmenopausal health and disease. To identify relevant literature, a comprehensive search was performed in the electronic database PubMed and Web of Science. The search covered articles published from January 2000 to September 2025 to capture the modern era of microbiome research.

The search strategy employed a combination of the following key terms and Boolean operators: (“postmenopause” OR “postmenopausal”) AND (“microbiome” OR “microbiota”) AND (“gut” OR “vaginal” OR “urinary” OR “urogenital”) AND (“osteoporosis” OR “cardiovascular disease” OR “metabolic syndrome” OR “genitourinary syndrome of menopause” OR “dysbiosis”).

Inclusion criteria encompassed: (1) original research articles and review articles; (2) studies conducted in postmenopausal human populations or relevant animal models; (3) articles focusing on the interplay between microbiota and postmenopausal conditions. Exclusion criteria included: (1) studies not published in English; (2) articles focusing solely on premenopausal populations without postmenopausal stratification.

This review was conceptualized and reported in line with the SANRA guidelines, ensuring a structured and quality-focused approach.

### 2.1 Postmenopausal microbiome dynamics and clinical implications

#### 2.1.1 Vaginal microbiome (VM) remodeling

The postmenopausal VM undergoes profound structural remodeling, primarily characterized by a decline in Lactobacillus dominance, a genus critical for maintaining a low pH environment and epithelial integrity. This community shift often results in an increase in alpha diversity metrics within some populations, a state that has been associated with elevated vaginal pH and a higher prevalence of conditions such as bacterial vaginosis and recurrent urinary tract infections (Chen et al., 2025; Kim et al., 2021; Shardell et al., 2021; Ouarabi et al., 2021; Yoshikata et al., 2022a,b; Hugenholtz et al., 2022; Gliniewicz et al., 2019; Murphy et al., 2019). The primary mechanistic driver is persistent estrogen deficiency, which impairs glycogen deposition in the vaginal epithelium, thereby limiting the primary metabolic substrate for Lactobacillus (Smith and Ravel, 2017). Furthermore, the resulting dysbiotic state is recognized for its capacity to propagate a localized inflammatory response (Liu R. et al., 2024; Jimenez et al., 2024; Supriya et al., 2024). The heterogeneity in alpha-diversity changes post-menopause, as highlighted in Table 1, underscores that findings are dependent on the study population and methodology employed.

**Table 1:** Heterogeneity in VM diversity and clinical associations in postmenopausal women.

**References**	**Study design**	**Population characteristics**	**Alpha-diversity trend**	**Key clinical associations**
Shardell et al. (2021)	Longitudinal cohort	750 women (age 35–60); 2,111 person-visits	Shannon Index: CST-I: Low CST-IV-A: High	Higher Diversity ↔ Vaginal dryness CST-IV ↔ Higher prevalence of vaginal atrophy *Lactobacillus* dominance ↔ Lower odds of urinary incontinence
Kim et al. (2021)	Cross-sectional	30 women (11 pre-, 19 post-menopausal)	Species Richness: Postmenopausal < Premenopausal Species Diversity: Postmenopausal > Premenopausal	Significant reduction in *Lactobacillus* abundance postmenopause (23.7% vs. 63.2%)
Chen et al. (2025)	Cross-sectional	40 women (mean age 51 ± 8)	Alpha Diversity: Significantly higher in postmenopausal women	CST IV prevalence: 70% (Postmenopausal) vs. 40% (Premenopausal)
Ouarabi et al. (2021)	Cross-sectional	100 women (50 pre-, 50 post-menopausal); healthy community cohort	Shannon Index: Significantly increased in postmenopausal group (*p* < 0.0001)	*Lactobacillus* (82.45% to 47.32%, *p* < 0.0001) CST-IV profile (54% vs. 18% in premenopausal) genera like *Gardnerella, Corynebacterium, Mycoplasma*
Yoshikata et al. (2022a)	Randomized controlled trial (Baseline)	70 women (35 pre-, 35 post-menopausal); clinic-based	Shannon Index: Significantly increased in postmenopausal women (0.77 vs. 1.92, *p* < 0.0001)	*Lactobacillus* (72% to 10%, *p* < 0.0001) Vaginal pH (4.37 to 5.89, *p* < 0.0001) Overactive bladder symptoms
Hugenholtz et al. (2022)	Cross-sectional	Pre-or post-menopausal women with/without rUTI; multi-ethnic & patient cohorts	Shannon Index: Significantly higher in postmenopausal controls vs. premenopausal controls (Urine & Vaginal, *p* < 0.05)	*Lactobacillus* (Vaginal: 66.3% to 10.3%, *p* < 0.001) BV-anaerobes, Gram-positive uropathobionts rUTI associations: trends of pathobionts in cases, modulated by menopausal status & clinical background
Gliniewicz et al. (2019)	Cross-sectional	45 women (15 Pre, 15 Post, 15 Post+HT); grouped by pH & atrophy score	Community State: Postmenopausal women predominantly in high-diversity, non-*Lactobacillus* clusters (e.g., Cluster D)	*Lactobacillus* dominance (Pre: 8/15 vs. Post: 2/15) Vaginal pH & atrophy scores in high-diversity clusters HT effect: POST+HT microbiome resembles PRE (10/15 lactobacilli-dominated)
Murphy et al. (2019)	Cross-sectional	141 women (71 Pre, 70 Post); stratified by HIV status	Alpha Diversity: Significantly increased in HIV+ postmenopausal women	*L. crispatus* & *L. iners* in postmenopausal women (effect most pronounced in HIV+ group) *Gardnerella* & *Atopobium vaginae* in postmenopausal women Functional Impact**:** *E. coli* bactericidal activity in HIV+ postmenopausal women

This table summarizes findings from diverse studies to illustrate the heterogeneity in reports of postmenopausal vaginal microbiome restructuring. The specific direction of change in alpha-diversity metrics is not uniform and is highly dependent on the study population and methodological approaches. The findings presented represent associations, and causation should not be inferred. Reported changes in α-diversity metrics are highly sensitive to methodological approaches (e.g., sequencing depth, bioinformatic pipelines), and findings should be interpreted in this context. Findings: Consistent across most studies. Heterogeneous: Reported findings vary across studies, often due to differences in population or methodological approaches.

Beyond the decline of *Lactobacillus*, the postmenopausal vaginal microbiome undergoes broader ecological remodeling characterized by increased prevalence of facultative and strict anaerobes such as *Gardnerella, Prevotella, Streptococcus*, and *Corynebacterium*. This shift in community composition contributes to elevated vaginal pH and creates a pro-inflammatory environment that compromises mucosal barrier integrity.

#### 2.1.2 Gut microbiome (GM) metabolic-immune dysregulation

Postmenopausal GM restructuring features remodeled community composition, commonly characterized by diminished phylogenetic diversity and enrichment of taxa linked to metabolic dysregulation in several studies (Huang et al., 2017; Flores et al., 2012; Parida and Sharma, 2019; Kwa et al., 2016). Postmenopausal GM alterations are associated with reduced short-chain fatty acids (SCFAs) levels, which may compromise intestinal epithelial barrier integrity and contribute to disrupted immune homeostasis (Kau et al., 2011; Yao et al., 2023). The estrobolome is defined as the collection of genes within the gut microbiota capable of metabolizing estrogen. Estrogen is metabolized from its conjugated form to the deconjugated form through microbial β-glucuronidase (Plottel and Blaser, 2011). The GM modulates estrogen homeostasis through bacterial β-glucuronidase activity, reactivating estrogen metabolites that influence inflammation and lipid metabolism. Targeted GM interventions may restore eubiosis.

#### 2.1.3 Urinary microbiome (UM)

##### 2.1.3.1 Pathological significance

Postmenopausal UM exhibits a remodeled community composition, typically marked by depletion of protective Lactobacillus and enrichment of facultative pathogens (e.g., *Escherichia, Klebsiella*) (Magistro and Stief, 2019; Neugent et al., 2020; Javan Balegh Marand et al., 2021; Kroon et al., 2018). This compromises colonization resistance, increasing susceptibility to UTIs and bladder disorders (Govender et al., 2019; Chattrakulchai et al., 2025). Estrogen deficiency impairs urothelial homeostasis by reducing glycogen availability and antimicrobial defenses. This will also alter the composition of the microbiome in the urinary tract, further reducing natural resistance to infections and other diseases. It is important to note that studies of the UM require rigorous contamination controls due to its low-biomass nature, which includes the use of negative controls and specialized bioinformatic filters.

##### 2.1.3.2 Methodological considerations and contamination control

The analysis of the UM presents unique challenges due to its low microbial biomass, making findings highly susceptible to contamination from DNA extraction kits, reagents, and the laboratory environment. Therefore, a critical appraisal of the methodological rigor employed in primary literature is essential for interpreting reported findings. Key considerations for robust UM studies include the implementation of end-to-end negative controls, the application of bioinformatic decontamination tools, and the use of EQUC cross-validated with 16S/shotgun. A minimum reporting checklist summarizing these critical safeguards is provided in Supplementary Table S1.

### 2.2 Mechanistic frameworks of microbiome influence

#### 2.2.1 Vaginal-urinary microbiome pathophysiology

Substantial evidence suggests a significant concordance between the urinary and VM (Thomas-White et al., 2018; Brown et al., 2021; Robinson et al., 2019). Multiple studies have established correlations between the composition of the vaginal microbiota and urogenital symptomatology, including specific urinary incontinence phenotypes and recurrent cystitis (Shardell et al., 2021; Thomas-White et al., 2020; Lewis and Gilbert, 2020; Sekito et al., 2023). Furthermore, a prospective study indicates that the preoperative VM can significantly predict the likelihood of postoperative urinary tract infections (Occhino et al., 2024). The efficacy of vaginal estrogen therapy in modifying the vaginal microbiota and its association with a reduced incidence of UTIs has been demonstrated in several studies (Raz and Stamm, 1993; Ferrante et al., 2021; Anglim et al., 2022). However, the association is not universal; one study concluded that while the vaginal microbiota was linked to sexual pain in genitourinary syndrome of menopause (GSM), it showed no significant association with other urinary tract symptoms (Waetjen et al., 2023), highlighting the complexity of these interactions. Current research in postmenopausal women primarily focuses on correlations between the VM and genitourinary symptoms like UTIs, while direct investigations into the urinary tract microbiome itself remain less explored. Consequently, the mechanisms through which the vaginal microbiota influences urinary health are still unclear, with potential pathways including direct microbial translocation to the urethra or indirect effects mediated by hormonal and inflammatory pathways.

#### 2.2.2 Gut-bone axis and VM interplay

Postmenopausal osteoporosis is a systemic metabolic bone disorder fundamentally driven by estrogen deficiency, with the GM acting as a key mediator through the gut-bone axis (Ensrud and Crandall, 2017; Lu et al., 2021; Zaiss et al., 2019; Li et al., 2016).

Evidence from animal models has elucidated several mechanistic pathways. The “brain-gut-bone” axis has been identified, wherein neuropeptide Y mediates gut dysbiosis, increases intestinal permeability and systemic LPS, and ultimately promotes osteoblastic pyroptosis and bone loss—effects reversible by Y1 receptor antagonism and transmissible via FMT (Chen et al., 2023). Furthermore, gut microbiota-derived SCFAs contribute to bone homeostasis through their potent anti-inflammatory actions and by modulating key immune cells, such as by balancing Th17/Treg differentiation to control pro-osteoclastic signaling (Furusawa et al., 2013; Guo et al., 2023). Other pathways involving bile acid receptors (e.g., TGR5) and systemic inflammatory factors also play important roles in bone metabolism (Guan et al., 2023).

In human observational studies, associations have been established between altered GM composition, functional shifts (such as reduced SCFAs-producing capacity), and decreased bone mineral density in postmenopausal women (He et al., 2020). A preliminary association has also been reported between GM and osteoporosis, suggesting potential cross-systemic interactions, although this area remains underexplored (Yang et al., 2022). However, a Mendelian randomization analysis provides genetic evidence supporting a potential causal role of gut microbiota in bone loss, identifying specific bacterial taxa, such as the *Burkholderiales* order, as potentially influential (Xiao et al., 2024).

Notably, results from human interventional trials show the intervention altered the interactional network of gut microbiota, particularly among short-chain fatty acid-producing bacteria (Zhao et al., 2023). It significantly increased the expression of genes encoding components of carbohydrate metabolic pathways—including ABC transporters, the phosphotransferase system, and fructose and mannose metabolism—as well as choline-phosphate cytidylyltransferase.

Despite substantial mechanistic insights, a significant knowledge gap remains. The role of key mediators like SCFAs in postmenopausal women is largely inferred from animal studies (Chen et al., 2023; Furusawa et al., 2013; Guo et al., 2023; Guan et al., 2023), and the causal relationships in human populations require further validation.

#### 2.2.3 Microbiome-metabolite-estrogen networks

The gut microbiota regulates systemic estrogen levels through microbial β-glucuronidase activity, which catalyzes the deconjugation of estrogen metabolites in the enterohepatic circulation, facilitating their reabsorption into the bloodstream (Plottel and Blaser, 2011; Rietjens et al., 2017). The functional capacity of this system, collectively termed the estrobolome, can be assessed through specific measurable targets including: Fecal β-glucuronidase enzymatic activity (Cross et al., 2024; Honda et al., 2024), Quantification of bacterial *gus* gene families encoding this enzyme and comprehensive fecal and serum estrogen metabolite profiling. In postmenopausal women, it is hypothesized that an altered estrobolome may perturb residual estrogen metabolism, potentially influencing the compositional landscape of both the vaginal and urethral microbiomes (Yoshikata et al., 2022a). However, direct causal evidence linking estrobolome activity to clinical outcomes in peri/postmenopausal populations remains limited. Intriguingly, certain *Lactobacillus* species also possess β-glucuronidase activity, suggesting potential for localized estrogen metabolism within the urogenital tract. Nevertheless, this putative local regulatory function currently lacks direct experimental validation and represents a compelling avenue for future research.

Therapeutically, strategies targeting β-glucuronidase inhibition—through specific dietary components or microbial modulation—emerge as translational prospects rather than established clinical recommendations. These approaches warrant further investigation to determine their efficacy in managing postmenopausal health via precise manipulation of the gut-microbiome-estrogen axis.

#### 2.2.4 The gut-endothelial axis

**Figure 1:**
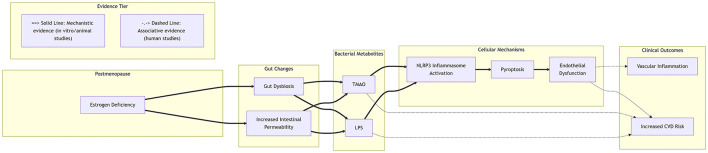
Potential mechanistic pathways linking postmenopausal gut microbiota dysbiosis to endothelial dysfunction. This schematic diagram illustrates one potential pathway by which postmenopausal estrogen deficiency may lead to gut microbiota dysbiosis and increased intestinal permeability, thereby promoting the systemic circulation of bacterial metabolites such as TMAO and LPS. These metabolites may activate the NLRP3 inflammasome pathway in vascular endothelial cells, triggering pyroptosis and subsequent endothelial dysfunction. It should be noted that this diagram represents only one potential mechanism described in this paper and omits many intermediate substances. Evidence levels distinguish two pathways: those supported by mechanistic evidence from experimental models (solid lines) and those supported by associative evidence from human observational studies (dashed lines), where causality remains unclear.

Accumulating evidence suggests gut dysbiosis may contribute to endothelial dysfunction through bacterial metabolites, though human evidence remains primarily associative. Studies demonstrate that gut-derived TMAO and LPS can activate the NLRP3 inflammasome (Chen et al., 2017; Zhang et al., 2020), triggering endothelial pyroptosis (Li et al., 2019; Arioz et al., 2019; Huang et al., 2020). Estrogen deficiency may promote this pathway by increasing gut permeability and systemic LPS translocation (Cristofori et al., 2021). As delineated in Figure 1, these human associations (dashed lines) do not establish causality for the mechanistic pathway (solid lines) observed in experimental models.

Observational studies consistently show correlations between elevated circulating TMAO or LPS levels and postmenopausal osteoporosis (Ma et al., 2024). However, these associations do not establish causality due to potential confounding factors, and human causality for this pathway remains uncertain. Direct evidence from human trials targeting this axis remains scarce. In summary, this axis represents a mechanistically plausible but clinically unproven pathway linking gut health to vascular outcomes in postmenopausal women.

### 2.3 Cross-system microbiome interactions in postmenopause

Such cross-niche interactions embody the core concept of Postmenopausal Microbiota Network Medicine. Microbial translocation across anatomical compartments represents a significant pathway influencing postmenopausal health through complex ecological interactions. Accumulating evidence indicates that gut-derived microbes can disseminate to distal sites, including the pulmonary and dermal systems, where they may modulate local immune responses and tissue homeostasis (Winkler and Thackray, 2019; McPherson et al., 2021). Animal studies of the gut-lung axis exemplify this phenomenon, demonstrating that gut metabolites can directly regulate pulmonary immunity and inflammatory cascades and may thereby influence respiratory pathophysiology under conditions of modeled estrogen deficiency (Perez Umana et al., 2025). Such interdependence necessitates integrated research frameworks to advance targeted interventions for postmenopausal disorders.

In parallel, gut-derived microbial metabolites facilitate essential cross-organ communication that contributes to physiological balance during postmenopause. SCFAs, key microbial metabolites, help maintain intestinal barrier integrity, modulate systemic inflammation, and circulate to exert pleiotropic effects on distal organs such as the liver, brain, and adipose tissue (Liu H. et al., 2024; Wang et al., 2023). SCFAs demonstrate dual regulatory capacity: orchestrating immune responses through histone deacetylase inhibition while regulating energy homeostasis via G-protein-coupled receptor activation, revealing therapeutic potential for metabolic complications characteristic of estrogen deficiency (Li et al., 2018; Tan et al., 2023; Du et al., 2024). The gut-liver axis dysfunction exemplifies this paradigm, where dysbiosis-induced metabolite perturbations exacerbate hepatic pathologies and propagate systemic inflammation in postmenopausal states (Xiang et al., 2021; Chu et al., 2025).

To critically appraise the current evidence and guide future clinical translation, we have synthesized the findings into an evidence matrix (Table 2) that aligns interventions with key clinical endpoints and grades their strength based on guideline recommendations and systematic reviews.

**Table 2:** Evidence matrix for microbiome-targeted interventions in postmenopausal.

**Intervention**	**rUTI recurrence**	**GSM symptoms**	**BMD/Bone turnover**	**Metabolic markers**	**CVD surrogates**
Vaginal estrogen (Kaufman et al., 2025)	**Recommended** (Kranz et al., 2024; Ackerman et al., 2025)	**Recommended**	Not Indicated	Not Indicated	Not Indicated
Probiotics (Ribeiro et al., 2025; Xiong et al., 2025)	**Context-dependent** (strain/dose/population sensitive)	**Context-dependent** (strain/dose/population **sensitive)**	**Context-dependent** (strain/dose/population sensitive)	**Context-dependent** (strain/dose/population sensitive)	**Context-dependent** (strain/dose/population sensitive)
FMT (Cross et al., 2024; Chen et al., 2023; Rico-Caballero et al., 2025; Palou et al., 2013)	**Exploratory**	**Exploratory**	**Exploratory**	**Exploratory**	**Exploratory**
Metabolite supplementation (Bhardwaj et al., 2025)	**Exploratory**	**Exploratory**	**Exploratory**	**Exploratory**	**Exploratory**
Enzyme inhibitors (Lv et al., 2025; Abbasi et al., 2025)	**Exploratory**	**Exploratory**	**Exploratory**	**Exploratory**	**Exploratory**

Evidence Strength Legend**: Recommended:** Supported by major clinical guidelines and robust, replicated RCT evidence. **Context-dependent:** Evidence of efficacy is mixed or demonstrably dependent on specific factors; not yet a standard recommendation. **Exploratory:** Primarily supported by preclinical or mechanistic data; clinical evidence in postmenopausal populations is absent or in very early stages. **Not Indicated:** The intervention is not intended or approved for this endpoint.

Evidence consistency: Recommendations are based on consistent findings from multiple high-quality studies. Context-dependent or exploratory classifications indicate greater heterogeneity or limited evidence.

These examples of gut-lung and gut-skin axes illustrate the broader principle of systemic microbial translocation and metabolite signaling, which underpins the more localized but equally critical interactions within the gut-vaginal-urinary network in postmenopausal women.

### 2.4 Future research imperatives for postmenopausal microbiome science

A deep understanding of the microbiota's role in the pathophysiology of postmenopausal disorders is crucial for advancing clinical management. Substantial evidence indicates that sustained estrogen deficiency enables the GM to actively modulate host inflammatory cascades and hormonal responses. This resultant dysbiosis is increasingly recognized as a significant contributor to the development of osteoporosis, cardiovascular disease, and metabolic syndrome. The bidirectional crosstalk between gut microbiota and host undergoes substantial disruption under estrogen-deficient conditions, leading to disturbed metabolic homeostasis. These insights suggest that microbiome-targeted interventions may offer promising avenues for restoring physiological balance and reducing disease risks.

Future research should prioritize the identification of key functional microbial taxa and their bioactive metabolites that regulate endocrine-inflammatory pathways. Concurrent investigation into host genetic and epigenetic factors influencing microbiome-host interactions is equally important. Achieving a comprehensive mechanistic understanding will require integrated approaches combining microbiology, endocrinology, and genetics to develop clinically relevant, predictive disease models.

Technological innovation remains indispensable for deciphering these complex relationships. Advances in metagenomic sequencing now enable comprehensive profiling of microbial communities during estrogen deficiency. The integration of machine learning with multidimensional datasets holds potential for identifying microbiome-based predictive signatures of disease susceptibility, possibly enabling preemptive interventions. Additionally, wearable devices offer opportunities for enhanced real-time physiological monitoring. The successful implementation of these technologies into clinical workflows is essential for advancing postmenopausal health management.

Despite substantial mechanistic insights and technological progress, significant translational barriers continue to hinder clinical application. Regulatory complexities and the extensive clinical validation required for microbiome-targeted therapies present considerable challenges. Considerable interindividual variation in microbiome composition further complicates the development of universal treatment protocols. Overcoming these obstacles demands coordinated efforts among researchers, clinicians, and regulators to establish standardized efficacy and safety frameworks while developing adaptive regulatory pathways. Strategic academia-industry partnerships will be crucial for accelerating the clinical implementation of microbiome-based interventions.

## 3 Conclusion

We now recognize that postmenopausal microbiome shifts act as critical pathophysiological mediators. Progressive dysbiosis shows clear mechanistic links to osteoporosis, cardiovascular disease, metabolic syndrome, and genitourinary disorders. This synthesis positions persistent microbial dysregulation as a central driver of degenerative processes—primarily through disrupted host metabolism, chronic inflammation, and endocrine dysfunction. These insights move beyond symptomatic management to reveal underlying etiological pathways.

Microbiome-targeted interventions hold genuine promise for reducing postmenopausal disease risks. Success requires phenotype-stratified approaches; for example, suppressing urease-producing pathobionts while restoring *Lactobacillus*-dominated communities to recalibrate dysbiotic networks.

Yet clinical implementation faces limitations: heterogeneous protocols, inadequate cohort sizes, and persistent mechanistic-translational gaps. Future research should prioritize longitudinal validation of microbiome-based strategies while developing personalized modulation frameworks that account for interindividual variability. Integrating these evidence-based approaches could meaningfully curb disease progression and healthcare burdens.

Ultimately, incorporating microbiome diagnostics into standard clinical assessments—and deciphering host-microbe crosstalk during chronic estrogen deficiency—will catalyze innovative therapeutics for this degenerative phase.

## 4 Discussion

The decline in estrogen levels that occurs naturally during the menopausal transition can precipitate a substantial and interconnected dysbiosis, encompassing the microbiomes of the gastrointestinal, vaginal, and urinary tracts. Rather than being considered a secondary symptom, this dysbiosis is emerging as a central pathogenic driver that propagates inflammation, metabolic dysfunction and specific tissue pathologies (e.g., urogenital epithelial atrophy, bone loss, vascular endothelial dysfunction), thereby accelerating the onset of chronic diseases that define the postmenopausal syndrome.

A significant finding is the demonstration of functional interdependence among microbial niches. The demonstrated concordance between vaginal and UM suggests the presence of a shared ecosphere in which dysbiosis in one compartment readily destabilizes the other, increasing susceptibility to conditions such as recurrent urinary tract infections. However, the underlying mechanisms—whether involving direct microbial translocation, impaired immune signaling, or local endocrine changes—remain inadequately elucidated. The existence of reported contradictions, such as the absence of a clear association between vaginal microbiota and certain urinary symptoms, underscores the complexity of these ecosystems and cautions against the making of generalized assumptions of causality.

It is also crucial to acknowledge the methodological limitations inherent in studying the UM. As a low-biomass environment, findings in this field are particularly vulnerable to contamination. While we have focused on studies that employ stringent controls (as outlined in the Methodological Considerations subsection), the interpretation of UM data across the literature must be tempered by an awareness of these technical challenges. Future studies should adhere to standardized reporting checklists to improve reproducibility and comparative analysis.

In a similar manner, the gut-bone axis signifies an additional pivotal pathway through which systemic influence is exerted. Whilst there is a general consensus that microbial metabolites, such as short-chain fatty acids, modulate bone metabolism through immunoregulatory mechanisms, this consensus is heavily dependent on findings from animal studies. The paucity of human research, particularly that focusing on postmenopausal populations, signifies a substantial knowledge gap. The preliminary association between vaginal dysbiosis and osteoporosis introduces an intriguing yet underexplored possibility, namely the hypothesis that local genitourinary microbiota may exert indirect effects on systemic processes such as bone.

The considerable heterogeneity observed in the outcomes of microbiota-targeted interventions highlights the critical influence of several key effect modifiers. The efficacy of any given approach is unlikely to be universal but is instead modulated by host and intervention-specific factors. Notably, the stage of menopause may determine the plasticity of the microbial ecosystem and the host's physiological responsiveness. Host characteristics such as baseline BMI and microbiota composition create a unique metabolic and ecological context that can dictate the success of an intervention. Furthermore, the effects of probiotics are highly strain-specific, and variables such as optimal dosage and treatment duration for achieving persistent effects in the postmenopausal population remain to be fully elucidated. Future research and clinical translation must therefore move beyond a one-size-fits-all model and adopt a stratified approach that accounts for these variables to identify the populations most likely to benefit.

Therapeutic strategies that are centered on the microbiome—including probiotic consortia, FMT, and metabolite supplementation—show great promise but must be contextualized within a framework of precision medicine. The efficacy of vaginal estrogen therapy in modifying microbial composition and reducing infection risk highlights the importance of targeting the estrogen-deficient phenotype. In view of the considerable interindividual variability in microbiome composition, the attainment of future success is likely to be contingent on stratified approaches that take into account personal biomarkers and microbial profiles. Furthermore, the cross-system nature of postmenopausal dysbiosis suggests that interventions that simultaneously address the gut and urogenital compartments may yield synergistic benefits, surpassing the outcomes of localized treatments.

This review also exposes important limitations in the current evidence. A significant proportion of studies are correlative, and human data are frequently derived from small or heterogeneous cohorts. While the utilization of animal models is undoubtedly beneficial in the formulation of mechanistic hypotheses, there remains the possibility that such models do not fully recapitulate the intricacies of human postmenopausal physiology. In order to address these gaps, future research should prioritize large longitudinal human studies that can track microbiome dynamics relative to disease onset. Mechanistic studies employing advanced tools, including multi-omics integration, gnotobiotic models, and tissue-on-chip technologies, will be essential to validate causal pathways and identify novel therapeutic targets. Moreover, translational efforts must overcome challenges related to regulatory standards, reproducibility, and individualized treatment protocols. Collaborative frameworks involving researchers, clinicians, and industry partners will be crucial in accelerating the development of safe and effective microbiome-based therapies.

In summary, the microbiome plays a dynamic and influential role in postmenopausal health and disease. The application of the multisystem dysbiosis concept to the understanding of menopause has the potential to engender a paradigm shift in the way its multifarious pathologies are perceived. Despite the existence of significant scientific and translational challenges, the current evidence supports the integration of microbiome-targeted strategies into future therapeutic approaches. By elucidating the precise dialogues between microbes and the host, we can advance toward personalized interventions capable of mitigating the degenerative consequences of estrogen deficiency and improving the healthspan of postmenopausal women.
