# Supplementary material for: Multi-site microbiota crosstalk in the postmenopausal: from dysbiosis mechanisms to precision interventions
Source: Front Microbiol. 2026 Feb 19;17:1702700. doi: 10.3389/fmicb.2026.1702700 (PMC12960634; doi:10.3389/fmicb.2026.1702700)
Supplement: Supplementary file 1 [file Table_1.docx]

**Table S1: Focused Quality Control Checklist for Urinary Microbiome Studies**

| Study Citation | Sampling Pathway | EQUC Cross-validated with 16S/Shotgun | End-to-End Negative Controls | Decontam Processing | LOD Thresholds | qPCR Biomass/ Spike-in | Batch Effects Control |
| --- | --- | --- | --- | --- | --- | --- | --- |
| Study1 (1) | Y | N | Y | P | Y | N | N |
| Study2 (2) | Y | N | Y | Y | Y | N | N |
| Study3 (3) | Y | Y | N | N/A | Y | N | N |
| Study4 (4) | Y | N | Y | Y | Y | N | N |
| Study5 (5) | P | N | N | N/A | N | N | N |
| Study6 (6) | Y | N | P | Y | Y | N | N |

**Table S1: Purpose:** This checklist specifically evaluates methodological quality of urinary microbiome studies based on key technical criteria for low-biomass samples.

**Scoring Guide:** Y (Yes) = Clearly described and appropriately implemented; P (Partial) = Mentioned but insufficiently described; N (No) = Not mentioned or inappropriate; N/A = Not applicable. **Note:** This checklist was developed with reference to the STORMS (Strengthening The Organization and Reporting of Microbiome Studies) reporting guideline(7).

1. Shardell M, Gravitt PE, Burke AE, Ravel J, Brotman RM. Association of Vaginal Microbiota With Signs and Symptoms of the Genitourinary Syndrome of Menopause Across Reproductive Stages. The journals of gerontology Series A, Biological sciences and medical sciences. 2021;76(9):1542-50.

2. Brown SE, Robinson CK, Shardell MD, Holm JB, Ravel J, Ghanem KG, et al. Assessing the Concordance Between Urogenital and Vaginal Microbiota: Can Urine Specimens Be Used as a Proxy for Vaginal Samples? Frontiers in cellular and infection microbiology. 2021;11:671413.

3. Thomas-White K, Taege S, Limeira R, Brincat C, Joyce C, Hilt EE, et al. Vaginal estrogen therapy is associated with increased Lactobacillus in the urine of postmenopausal women with overactive bladder symptoms. American journal of obstetrics and gynecology. 2020;223(5):727.e1-.e11.

4. Occhino JA, Byrnes JN, Wu PY, Chen J, Walther-Antonio MR. Preoperative vaginal microbiome as a predictor of postoperative urinary tract infection. Scientific reports. 2024;14(1):28990.

5. Ferrante KL, Wasenda EJ, Jung CE, Adams-Piper ER, Lukacz ES. Vaginal Estrogen for the Prevention of Recurrent Urinary Tract Infection in Postmenopausal Women: A Randomized Clinical Trial. Female pelvic medicine & reconstructive surgery. 2021;27(2):112-7.

6. Anglim B, Phillips C, Shynlova O, Alarab M. The effect of local estrogen therapy on the urinary microbiome composition of postmenopausal women with and without recurrent urinary tract infections. International urogynecology journal. 2022;33(8):2107-17.

7. Mirzayi C, Renson A, Zohra F, Elsafoury S, Geistlinger L, Kasselman LJ, et al. Reporting guidelines for human microbiome research: the STORMS checklist. Nature medicine. 2021;27(11):1885-92.
